# Knowledge of Intravenous Cannulation among Interns of a Teaching Hospital: A Descriptive Cross-sectional Study

**DOI:** 10.31729/jnma.7222

**Published:** 2022-03-31

**Authors:** Mona Sharma, Sushil Paudel, Ujma Shrestha, Bikash Sitaula

**Affiliations:** 1 Department of Anesthesiology, Kathmandu Medical College and Teaching Hospital, Sinamagal, Kathmandu, Nepal; 2 Department of Orthopedics and Trauma Surgery, Institute of Medicine, Tribhuvan University Teaching Hospital, Maharajgunj, Kathmandu, Nepal

**Keywords:** *complications*, *cannulation*, *intravenous*, *knowledge*

## Abstract

**Introduction:**

Health care professionals are expected to have a basic understanding of all procedures performed on the patient. Their knowledge has direct implications on patients' morbidity. Interns perform intravenous cannulation during their clinical rotation, their awareness about the procedure are unknown. The aim of this study was to find the knowledge of intravenous cannulation among the interns of the teaching hospital.

**Methods:**

A descriptive cross-sectional study was conducted in a teaching hospital from November 2020 to October 2021 after clearance from the Institutional Review Committee (Reference number: 2611202002). A total of 151 interns were taken using the convenience sampling method. We prepared a structured questionnaire. This was distributed among interns who had completed 6 months of internship. Data entry and analysis was done using Microsoft Excel. Point estimate at 95% Confidence Interval was calculated along with frequency and proportion for binary data.

**Results:**

Out of 151 interns, 84 (55.62%) (47.70-63.54 at 95% Confidence Interval) had knowledge about intravenous cannulation. Most of the interns 117 (77.48%) had knowledge about the appropriate place for cannulation. Awareness about serious complications of cannulation was poor; 106 (70.19%) had never heard of these terms. Handwashing was implied as important by 70 (46.36%).

**Conclusions:**

The knowledge about intravenous cannulation among interns of teaching hospitals was poor compared to the similar study done in a similar setting.

## INTRODUCTION

A peripheral intravenous (IV) cannula is a hollow catheter placed into a vein for the short-term administration of drugs, fluids, blood, electrolytes, and nutrition. Although a relatively easy and uncomplicated procedure, it can act as a high risk for infection by allowing direct microbial entry to the bloodstream from the traumatized skin and vein wall.^1^ Complications such as phlebitis, infiltration, and thrombus formation can increase morbidity and the length of hospital stay.^2^

These complications can be minimized by standard practice, education, adherence to proper sterility, proficiency, and familiarity with the procedure and equipment. Proper selection of vein, site, and catheter size according to the clinical need is undoubted.^3^

The aim of this study was to find the knowledge of IV Cannulation among the interns of the teaching hospital.

## METHODS

This descriptive cross-sectional study was conducted in Kathmandu Medical College and Teaching Hospital. The study was conducted from November 2020 to October 2021. Ethical approval taken from the Institutional Review Committee of Kathmandu Medical College (Reference number: 2611202002). A convenience sampling method was used to recruit interns for the study. All interns who had completed 6 months of training were included. The sample size was calculated using the formula:

n = (Z^2^ × p × q) / e^2^

  = (1.96^2^ × 0.5 × 0.5) / 0.08^2^

  = 151

Where,

n = minimum required sample size
Z = 1.96 at 95% Confidence Interval (CI)
p = prevalence taken as 50% for maximum sample size
q = 1-p
e = margin of error, 8%

A structured questionnaire was used to obtain information about the knowledge of peripheral intravenous cannulation. The questionnaire was validated with the help of experts. The experts were asked to rate the questions from 1 to 10. Questions that were marked less than six were not included in the questionnaire. Interns were included only after informed consent was obtained. The investigator distributed the questionnaire to all the study participants. The participants were instructed to fill all the answers accurately as per their knowledge. The data were entered in Microsoft Excel and statistical analysis was done using Excel. Narratives, tables, and charts were used to present data in numbers and percentages. Point estimate at 95% Confidence Interval was calculated along with frequency and proportion for binary data.

## RESULTS

Out of 151 interns, 84 (55.62%) (47.69-63.54 at 95% Confidence Interval) had knowledge about IV cannulation. Most of the interns knew the appropriate veins for cannulation are in a non-flexion part of the upper extremity 117 (77.48%). Awareness about serious complications of cannulation such as extravasation and infiltration was poor; 106 (70.19%) interns had never heard of these terms. Sixteen gauge cannula was the most common answer 70 (46.35%) for choice of a cannula for shock.

**Figure 1:**
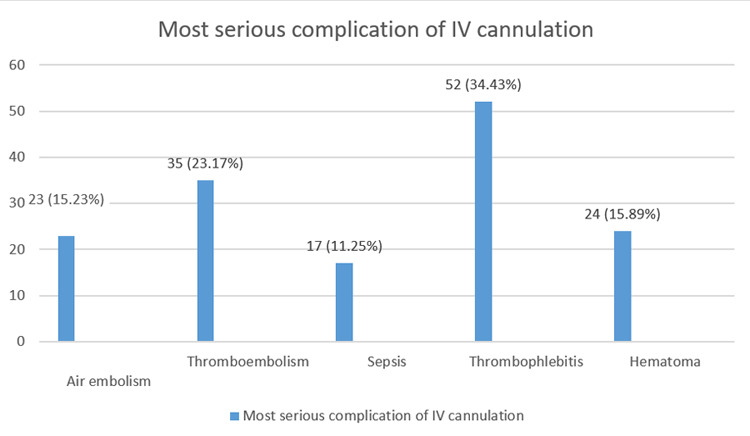
Complication of IV cannulation (n= 151)

The appropriate names of veins for cannulation were named by 70 (46.35%) whereas 52 (34.43%) had a blank response. On the query for the requirement of hand hygiene for IV cannulation, 23 (15.23%) did not think any hand hygiene was required. The most serious complication as per the questionnaire was thrombophlebitis 52 (34.43%) (Figure 1).

**Figure 2:**
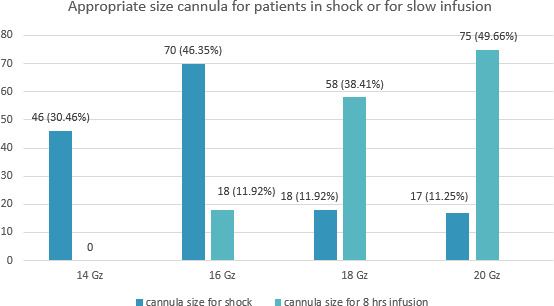
Appropriate size cannula for patients in shock or for slow infusion (n= 151).

When asked for the definition of phlebitis 110 (72.84%) could not answer. The most common size of the cannula for slow infusion of 8 hours was 20 gauge as per 75 (49.66%) interns (Figure 2).

According to our study group, most complications occur in the geriatric age group 116 (76.82%) and then the pediatric age group 35 (23.17%). On asking about heparin lock, 58 (38.41%) thought it was a cap, 41 (27.15%) had no idea and only 52 (34.43%) answered that it was a heparin flush to keep the catheter patent. Knowledge about hand hygiene is depicted below (Table 1).

**Table 1:** Knowledge of hand hygiene in interns (n= 151).

Hand hygiene	n (%)
Wash hands before cannulation	70 (46.36)
Wear gloves during cannulation	145 (96.02)

**Figure 3:**
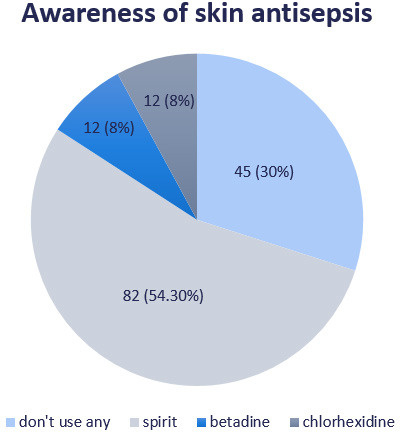
Awareness of skin antisepsis (n= 151).

Forty-six (30.46%) did not think that skin antisepsis was required (Figure 3).

## DISCUSSION

This study was conducted to know about the interns' knowledge about IV cannulation. As clinical practice consolidates and improves clinical knowledge and practice, we chose interns after six months of training. Intravenous catheters are usually performed in the distal part of the upper extremity. Forearm veins are the most suitable for cannulation as there is no flexion and provide a high surface area to secure. Further, it is reported that pain is lesser and the risk of accidental removal and occlusion is decreased.^4^ Our interns were knowledgeable about this fact 117 (77.5%). However, while naming the veins most were unaware 99 (65.6%). In a similar study conducted in India, 91.7% were knowledgeable about ideal IV cannula site and 74% could name an appropriate vein.^5^

An appropriate size of the cannula depends on the clinical scenario. A large gauge such as 14, 16 gauge is used in an emergency situation, where a large amount of fluid is to be administered in a short period of time, such as shock and a smaller gauge cannula is apt for cases where slow infusion is optimal. The small size also causes less resistance in the vessel and thus, lesser complications. Evidence suggests that catheter size has an impact on device survival rate. Large bore catheters such as 18 gauge or higher had a higher incidence of thrombosis and smaller bore cannula such as 22 gauge had a higher incidence of infiltration.^6^ Our interns were well versed, with these facts as 76.7% of them replied that they would use 14 and 16 gauge cannula for shock and 49.7% would use 20 gauge for infusion over eight hours. Size is also however dependent on other factors such as age, the viscosity of the drug, need for pressurized boluses. However, 20 gauge is suitable for adults for various applications according to studies.^7^ Similarly, studies conducted previously have cited that most interns have chosen 20 gauge cannula for slow infusion 47.2%^8^ and 62.4%.^5^

Many complications occur due to the IV cannula, though seemingly simple. Complications occur due to faulty technique, catheter occlusion, hematoma, thrombophlebitis, extravasation, infiltration, and catheter-associated bloodstream infection.^9,10^ One of the most common complications is thrombophlebitis. Only, 52 (34.4%) were aware of this complication. When the vein is simply inflamed it is known as phlebitis, but when complicated with thrombus, then it is thrombophlebitis. Very few of the interns could define phlebitis 41 (27.2%). The causes of thrombophlebitis can be trauma to the vessel wall, size of the cannula, infusate, and bacterial colonization.^11^ The most common bacterial flora causing thrombophlebitis is Staphylococcus aureus.^12^ Unlike our finding one study^8^ reported 60% of interns knew about phlebitis and another study showed 99% of participants^5^ were well versed with the topic.

Unintentional leaking of non-vesicant solution out of the vein is infiltration whereas leaking of the vesicant solution is extravasation.^13^ Vesicant solution can damage the tissue, and hence prompt action is required once infiltration or extravasation is identified. Our study population; 106 (70.3%) however, were not well versed with these complications. Likewise, a study showed that 50% of interns had no knowledge about infiltration or extravasation.^8^

Hand hygiene is one of the most important steps to reduce infection transmitted by health care professionals. The intravenous cannula should be performed by an aseptic non-touch technique. It is recommended to wash hands before and after the procedure. The most common answer to hand hygiene was to wear gloves 145 (96.2%) in our study. Similar to our data, another study^5^ reported 97.8% of interns in their study also responded that gloves during IV cannulation were essential. Unlike our 46.5% population who said handwashing was important, 94.4% and 72.4% of interns answered that handwashing was critical for infection control for IV cannulation in different studies.^5,8^

The limitation of this study was that it was specifically targeted on theoretical knowledge and their answers may be biased or inaccurate as they were answering for a faculty member and their actual practice may be different.

## CONCLUSIONS

The knowledge about IV cannulation among interns of teaching hospitals was poor compared to the similar study done in a similar setting.
